# The experience of long-term care staff caring for people with dementia in low- and middle-income Countries (LMICs): A qualitative evidence synthesis

**DOI:** 10.1177/14713012251346597

**Published:** 2025-05-28

**Authors:** Xiaoyang Li, Heather Wilkinson, Mengying Zhang, Sarah J. Rhynas

**Affiliations:** School of Health in Social Science, 3124 University of Edinburgh, UK; Edinburgh Centre for Research on the Experience of Dementia (ECRED), School of Health in Social Science, 3124 University of Edinburgh, UK; School of Health in Social Science, 3124 University of Edinburgh, UK; Nursing Studies, School of Health in Social Science, 3124 University of Edinburgh, UK; Edinburgh Centre for Research on the Experience of Dementia (ECRED), School of Health in Social Science, 3124 University of Edinburgh, UK

**Keywords:** dementia, dementia care, long-term care staff, long-term care settings, qualitative evidence synthesis, systematic review

## Abstract

**Background:** The demand for long-term residential care for people with dementia is, especially for those in the moderate to severe stages, increasing. However, dementia care services within long-term care (LTC) settings remain underdeveloped in low- and middle-income countries (LMICs). **Objectives:** This qualitative evidence synthesis aimed to explore LTC staff’s experiences of taking care of people with dementia in LTC facilities in LMICs. **Methods:** A comprehensive literature search was conducted of six databases in January 2023 and updated in August 2024 for qualitative studies of LTC staff’s experience of caring for people with dementia. Thematic synthesis was utilised for data synthesis, and NVivo facilitated this process. **Results:** 8,565 studies were screened, and 11 articles were included in this review. The studies included were conducted in Brazil (*n* = 2), China (*n* = 5), India (*n* = 1), Iran (*n* = 1), Malaysia (*n* = 1) and South Africa (*n* = 1) between 2012 and 2024. An overarching theme was identified: We are an island: underdeveloped dementia care within the LTC settings in LMICs, and the following categories identified as (1) the development of care provided for people living with dementia within care home settings is in its infancy; (2) the positive and effective coping strategies that may help embrace a brighter future of dementia care; (3) the deficiencies in caregiving approaches that contributed to poor-quality care for people living with dementia. **Conclusions:** The development of dementia care services within LTC in LMICs is still in its early stages. The main concern is the lack of available support and training for care staff and their insufficient dementia knowledge and care competencies. We hope that this review will help to increase attention to this significant issue of long-term institutional care for people with dementia in LMICs and that further research could investigate and enhance potential improvements in the practical implementation of long-term residential dementia care.

## Introduction

The global population is undergoing rapid ageing, and it is estimated that by the year 2050, approximately 80% of older people will reside in lower- and middle-income countries (LMICs) (World Health Organization, 2022). Dementia, as an intricate neurological disorder that is associated with the progressive deterioration of cognitive functions within the brain, presently stands as the seventh leading cause of mortality across all diseases and concurrently represents a major cause of disability and dependence among the demographic of older people worldwide (World Health Organization, 2021, 2024). There are estimated to be over 55 million people living with dementia globally, a large proportion of whom (61%) reside within the LMICs (World Health Organization, 2024).

The deterioration of the cognitive function of people with dementia leads to increasingly complex care and support needs, which are crucial to their quality of daily life (Terkelsen et al., 2020). In LMICs, about 90% of the care for those people living with dementia takes place in the home (Alzheimer’s Disease International, 2023), where formal care services for people with dementia are scarce (Seeher et al., 2023; World Health Organization, 2021). Thus, family caregivers undertake the primary role of taking care of their family with dementia. Along with the progression of dementia and impairment in functional abilities, such as mobility, personal hygiene and communication, people with dementia become increasingly dependent on others for assistance in activities of daily living (Slachevsky et al., 2019), and thus, bring challenges for the family caregivers (Abreu et al., 2020). Previous studies have shown that taking care of people with dementia at home requires significant time and effort from family caregivers and often comes at the expense of their own well-being and caregiving capacity (Hazzan et al., 2022). In the meantime, the concern about home care for people with dementia in LMICs is also related to a diminishing availability of family caregivers, which has been influenced by broader social transformation, including urbanisation, increased female labour participation, reduced household sizes and migration of younger generations (Fam et al., 2019). These societal changes are influencing traditional family-based caregiving structures and reducing the capacity of home-based care. Considering the challenges of home care for people with dementia and the family caregivers, as detailed above, triggers an increasing demand for support from long-term institutional care.

However, in most LMICs, long-term care (LTC) services remain underdeveloped and significantly constrained by limited resources, particularly in the field of dementia care (Prince et al., 2016; World Health Organization, 2021). A specific concern is the limited capacity of health and social care workers to provide dementia care, mainly due to a lack of continuous, high-quality training, which can negatively impact the quality of care provided for people living with dementia (Seeher et al., 2023; World Health Organization, 2021). Evidence suggests that education and training for care staff can improve the quality of care for people with dementia, encourage mental well-being and improve the work experience for caregivers in LTC settings (Briones-Peralta et al., 2020; Torres-Castro et al., 2022). Nevertheless, most evidence of effective training programmes comes from high-income countries. There is scarce evidence concerning interventions for providing support and training programmes for caregivers in LMICs (Salcher-Konrad et al., 2023). Findings from studies conducted in high-income countries may not be transferable to the distinct social and cultural environments and healthcare systems with limited resources in LMICs. Thus, there exists a requirement for evidence to explore the LTC staff’s care experience of people living with dementia in LMICs. This qualitative evidence synthesis was conducted to consolidate the fragmented body of primary research on LTC staff experience of people with dementia in LMICs, develop a comprehensive understanding of their perspectives and provide insights to inform culturally appropriate policy and practice.

### Why was it important to conduct this review

This review provides context-specific insights into the lived experiences of dementia care staff, which have the potential to support evidence-informed policy decisions, guide investment in workforce training, and shape sustainable long-term care models that reflect the specific needs and realities of under-resourced settings. By centring the voices of LTC staff, the findings of this review offer evidence that can support the design of responsive support systems and contribute to improving the quality of institutionalised dementia care and the experiences of LTC staff of people with dementia in these resource-constrained settings. In doing so, this review aligns with global calls to strengthen dementia care infrastructure through context-sensitive and equitable approaches.

## Aim

This synthesis of evidence aimed to understand care home staff’s experience of caring for people with dementia living in LTC settings in LMICs.

## Methods

This qualitative evidence synthesis applied the Preferred Reporting Items for Systematic Reviews and Meta-Analyses (PRISMA) guideline (Moher et al., 2009).

### Eligible care settings and participants

The LTC facilities included in the review referred to the care settings that deliver care services to support individuals with activities of daily living (ADLs) such as bathing, dressing, eating, toileting, and mobility assistance, as well as medical and nursing services if needed. Whether the LTC facilities are named residential facilities, nursing homes, care homes, or institutional care settings, the key characteristic of these care facilities is that the care services continue for prolonged periods. Thus, community-based short-term service centres did not meet the criteria for inclusion.

The inclusion criteria for ‘LTC staff’ were personnel whose work category was directly relevant to providing care for the daily living of people living with dementia, comprising caregivers, including nurses and care assistants, and other groups of support workers, for example, institution managers, activities coordinators, psychotherapists, and counsellors.

### Literature search and selection criteria

The search strategy was developed with the assistance of an academic librarian, and it was discussed and reached a consensus among the review team. We applied the Sample, Phenomena of Interest, Design, Evaluation, and Research (SPIDER) framework (Cooke et al., 2012) to define the review question and identify relevant search terms. The Medical Subject Headings (MeSH), synonyms and spelling differences of each search term were combined during searches. A comprehensive search was conducted of the major biomedical, nursing and social science databases in January 2023, including CINAHL Plus (for nursing and allied health), MEDLINE and EMBASE (for clinical and biomedical literature), PsycINFO (for psychological and mental health aspects), Web of Science Core Collection and Scopus (broad multidisciplinary coverage), and ASSIA (for applied social sciences). These databases were selected due to their extensive coverage of peer-reviewed literature relevant to health sciences, nursing, social care, and psychology, which are disciplines central to understanding dementia care. The search strategy was modified to suit each database. An updated search was conducted with the original search strategies in August 2024 to identify newly published studies since the initial search. The full search strategy is available in Appendix 1. The inclusion and exclusion criteria were summarised as shown in Table 1.

**Table 1.** Inclusion and exclusion criteria.

Inclusion criteria	Exclusion criteria
The included studies explore the experiences of LTC staff who work at the LTCFs and take care of people living with dementia	Studies from other health care settings (e.g., home care or acute hospitals) will be excluded
The included studies were conducted in LMICs according to the country classification by income of the World Bank (World Bank, 2024)	Studies exploring the experiences of people with dementia and their family caregivers will be excluded
The included studies were either qualitative or mixed methods with a qualitative component	Secondary studies such as literature reviews and systematic reviews will be excluded
The included studies were written in English and published in a peer-reviewed journal	Conference proceedings, commentary articles, thesis and dissertations will be excluded

### Study screening

The Covidence platform, a web-based collaboration software, facilitated the screening process (Covidence, 2024). Studies retrieved from the databases were uploaded to the Covidence, and duplicate records were automatically identified and removed. Two authors of this review independently screened the titles, abstracts, and full texts of the studies while adhering to the inclusion and exclusion criteria. Any disagreements of opinion were sorted out through discussion between the reviewers.

### Data extraction

A data extraction form was created in advance. It included the following fields: author, year of publication, country of publication, aims, sample, participant characteristics, care settings, data collection and analysis methods, and main findings/themes. The first review author independently extracted the information from the included studies and organised them within a standardised format to enable the quality assessment and evidence synthesis of the included studies.

### Quality appraisal

The quality assessment of all included studies was conducted using the Critical Appraisal Skill Programme (CASP) Qualitative Checklist (Critical Appraisal Skills Programme, 2018), specifically developed for qualitative studies. The CASP qualitative checklist comprises 10 questions, each addressing a unique methodological facet of a qualitative study regarding the research objective, methodology, research design, recruitment, data collection, data analysis, ethical issues, reflexivity, statement of findings and the value of the research.

### Data synthesis

Thematic synthesis (Thomas & Harden, 2008) was utilised as the method of data analysis, which is presented as a well-established approach that maintains a transparent connection between conclusions and the content of original studies and generates novel interpretations, concepts and hypotheses. The data extracted from primary studies for synthesis included all of the text under ‘results’ or ‘findings’ sections and were entered into the QSR NVivo software to facilitate coding.

## Findings

### Search outcome

**Figure 1.**
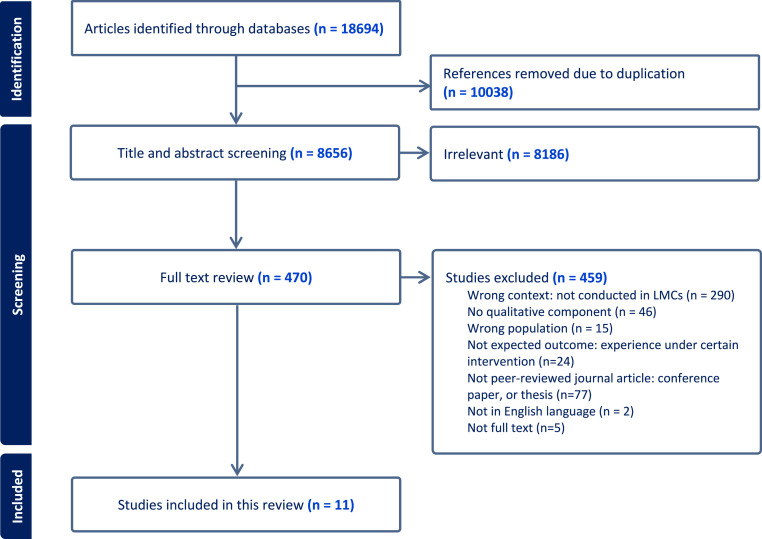
PRISMA flow chart for the literature screening (Moher et al., 2009).

In January 2023, 8 articles were eligible to be included in this review after initial searching of articles, screening of titles and abstracts and further review of the full texts. We carried out an updated literature search in August 2024, which includes relevant studies published between January 2023 and July 2024, to ensure the results of our review are up to date. We identified 935 new articles using the original search strategy. After screening and reviewing the articles, 3 articles were included and incorporated into the earlier data synthesis, updating the findings of this qualitative evidence synthesis. The screening process for the study was summarised by a PRISMA flow chart, presented in Figure 1.

### Characteristics of included studies

Included studies were conducted in Brazil (*n* = 2), China (*n* = 5), India (*n* = 1), Iran (*n* = 1), Malaysia (*n* = 1) and South Africa (*n* = 1) between 2012 and 2024. 200 participants were recruited across all studies, including 136 nurse assistants, 32 nurses, 26 institution managers, 3 physiotherapists, 2 social workers and 1 physician. Sixty-eight per cent of the participants were nurse assistants. The majority of the participants were over 40 years old. The gender of these participants was predominantly female. Except for 51 participants whose gender was not available (de Melo et al., 2023; Md Hussin et al., 2023; Shrestha & Tranvag, 2022; Yektatalab et al., 2012), there were 122 females and 27 males. For the participants’ average years of work experience, a large proportion had 2.5–5 years of work experience at LTC facilities. There was only one study where the recruited participants had worked in care facilities for an average of 15 years (van Wyk et al., 2017). A full overview of the characteristics of the included studies is presented in Appendix 2.

### Methodological quality

Two reviewers applied the qualitative study’s CASP checklist to the included studies and conducted a quality assessment. No studies would be excluded based on the quality appraisal; instead, the assessment was employed as a criterion for methodological rigour. All included studies scored 16 or more out of 20, indicating they were well-considered regarding methodological quality (see Appendix 3). Most studies did not adequately consider the relationship between the researcher and participants, indicating a deficiency in the researcher’s reflexivity that should merit a certain level of attention.

### Synthesised finding

Following the thematic synthesis approach proposed by Thomas and Harden (2008), the process involves three interrelated stages: line-by-line coding, the generation of ‘descriptive’ themes, and the development of ‘analytical’ themes. These stages are not strictly sequential and often overlap, allowing for a more dynamic and integrative approach to synthesising qualitative research. From the first step of line-by-line coding, the first reviewer temporarily put the review questions aside while staying close to the original study findings, generating a rich ‘bank’ of codes that almost covered all aspects of dementia care practices in LTC facilities. The ‘descriptive’ themes were grouped by comparing similarities and differences among the codes. Then, the ‘analytical’ themes regarding the review question and the understanding obtained through this cyclical and iterative process were identified. The embedded principle of staying ‘close’ and then ‘going beyond’ to the outcomes of the primary studies was applied along with the whole coding process (Thomas & Harden, 2008).

#### Overarching theme: we are an island: underdeveloped dementia care within the LTC settings in LMICs

The included studies all described the experience of LTC staff who provide care for people living with dementia in LTC settings in LMICs. Based on the synthesis of each aspect of the dementia care experience from the studies reviewed, an overarching theme was identified: ‘We are an island’: underdeveloped dementia care within the LTC settings in LMICs. The following themes and sub-themes of the overarching theme are shown in Table 2.

**Table 2.** Overview of themes: we are an island: underdeveloped dementia care within the LTC home settings in LMICs.

Themes	Sub-themes
The development of care provided for people living with dementia within care home settings in its infancy	Care home environment
	Care services and care model
	Perceived understanding of dementia
The positive and effective coping strategies that may help embrace a brighter future of dementia care	Personalised approach
	Building emotional bonds
	Work emotionally
	Group activities
The deficiencies in caregiving approaches that contributed to poor-quality care for people living with dementia	Care practices that overlook the self-care ability of people with dementia
	Unmet care needs of people with dementia during caregiving
	Need to provide care with dignity
	Inadequate management of the changed behaviours of people with dementia

#### The development of care provided for people living with dementia within care home settings is in its infancy

##### Care home environment

The hospital-like care home environment was observed in one of the studies conducted in China and is considered undesirable for long-term residential care, including shared bedrooms, inadequate lighting, excessive environmental noise and inappropriate colour schemes, and overcrowded dining areas (Zhao et al., 2021). The suboptimal environment in the care home adversely impacted the well-being of residents with dementia by limiting their social interactions. It is suggested that the care home environments be improved to become more dementia-friendly and home-like to facilitate the quality of life for residents (Zhao et al., 2021).

##### Care services and care model

From the illustrations of participants, the content of care services was mainly focused on meeting the basic needs of people living with dementia in daily care (Yektatalab et al., 2012; Zhao et al., 2021). In this regard, they labelled the care provided for people with dementia as ‘general care’ rather than ‘dementia care’. The most prominent characteristic of the ‘general care’ was that the care provided for people living with dementia was the same as the care they provided for other residents without dementia (Shrestha & Tranvag, 2022).

*‘Over here, there are many dementia cases. If we had better training, we could understand more about how to treat them and care for them. We do not have such special training for dementia cases, so we just provide general care for them as we do for other patients. If we had training, then we could better understand the behaviours and more.’ *(Shrestha & Tranvag, 2022, p. 6)

Besides, the task-oriented work pattern left the care staff to perceive fulfilling their demanding tasks efficiently as care priorities during caregiving (Wang et al., 2022). The findings of a study set in China elucidated that the ethos of dementia care within long-term residential facilities was profoundly influenced by the medical-oriented care model, meaning that the health conditions of residents with dementia were a priority (Zhao et al., 2021). The person-centred care (PCC) model, as a widely used and recommended care model for dementia in Western countries, was not commonly adopted within care home settings in LMICs. A study conducted by Wang et al. (2022) investigated the care staff’s understanding of PCC. The findings revealed a general lack of awareness among the staff, for instance:

*‘I imagined myself in their situation. I mean, I would ask myself, if you were him or her, what would you want? My goal is to treat them right. This is PCC in my eyes.’* (Wang et al., 2022, p. 41)

Two other studies investigated caregivers’ experience in delivering PCC practices, emphasising the individualised principle in that care providers carefully examine the individual experiences of residents with dementia and utilise that knowledge to provide daily care services, as shown in the following quotes (de Melo et al., 2023; Md Hussin et al., 2023).

*‘Knowing the preferences of the older people helps to guide and calm them in times of confusion.’* (de Melo et al., 2023, p. 49)

While the studies reviewed did not specifically address whether caregivers in LMICs received comprehensive training in PCC, the findings suggest that the absence of such training may contribute to its limited adoption. It is important to recognise that the absence of PCC in these settings does not reflect negligence but rather a gap in exposure and training opportunities for caregivers.

##### Perceived understanding of dementia

An overall poor knowledge of dementia was demonstrated by most of the participants. The participants of care staff from an explorative qualitative study conducted in India described their perception of dementia based on the common symptoms of dementia during caregiving, such as restlessness, forgetfulness, confusion and walking mystery (Strom et al., 2021). There were also misunderstandings of dementia among participants as they regarded dementia as a normal ageing progress (Zhao et al., 2021), as a psychiatric problem (Zhao et al., 2021), and could be cured and back to ‘normal’ again (Strom et al., 2021). Besides, treating people with dementia as children and communicating with them in an ‘elderspeak’ manner (a patronising speech pattern characterised by simplified vocabulary, slowed speech, exaggerated intonation, and diminutives typically used when speaking to older adults) during caregiving was repeatedly raised by some of the care staff (Md Hussin et al., 2023; Wang et al., 2022; Zhao et al., 2021). However, there was debate regarding whether such behaviour was appropriate. While some participants perceived it as a positive care strategy because it showed their patience with those living with dementia, others thought it to be inappropriate or disrespectful when interacting with people with dementia and that they should be treated with whole dignity as adults.

#### The positive coping strategies that may help embrace a brighter future of dementia care

##### Personalised approach

Although most participants were unfamiliar with formal PPC as conceptualised in Western societies, the personalised approach was an effective care strategy based on their daily care experience, which is important for addressing the challenging behaviours of people with dementia. A member of the care staff illustrates the following description:

*‘Each person (with dementia) is different, and each of them has their peace and rhythm.’* (Siewert et al., 2021, p. 7)

According to the majority of other carers, the most important component to successfully deal with the challenges of aggression, restlessness, and agitation associated with dementia and promoting communication and interaction during daily care was to take a personalised approach that involved knowing each resident with dementia’s background, lifestyle, hobbies, and preferences (de Melo et al., 2023; Siewert et al., 2021; van Wyk et al., 2017; Wang et al., 2022), for example:

*‘At the beginning when we encounter them, we will inquire about their medical history, social history as we would want to know what makes them reach this stage. We can avoid some sensitive questions which can trigger them to become sad or angry.’* (Md Hussin et al., 2023, p. 499)

##### Building emotional bonds

Another approach of building emotional bonds and good rapport with residents with dementia was repeatedly mentioned by the caregivers as a positive strategy during the caregiving experience. The study conducted by Siewert et al. (2021) showed that bonding could be a therapeutic possibility to deal with aggression and resistance among individuals with dementia. Meanwhile, through gaining trust and building a good rapport with residents with dementia, a more in-depth understanding of them could be obtained, strengthening the personalised care approach and boosting the quality of care. For example, it was adopted among caregivers to establish a trusting relationship and build emotional bonds to identify potential signals of pain and address the needs of residents living with dementia (Yang et al., 2024), as a staff member described their experience as follows:

*‘These residents usually stay for a long time, and we treat them like family. We establish a trusting relationship with each other, so we are very sensitive to any abnormal behaviors they may exhibit.’ *(Yang et al., 2024, p. 103)

In addition to the emotional bonds between caregivers and people with dementia, de Melo et al. (2023) described that facilitating the socialisation among residents with dementia with other residents within their institutional community was an important aspect of caring for them with comfort and spirituality, as one participant noted:

*‘Caring for the patient comfort, where he can ambulate and may be able to socialize with the other old residents for experience exchange. As well as work with their spirituality for life meaning. Remembering that this place may be their last house, reminding the patient of their condition is crucial for life quality.’ *(de Melo et al., 2023, p. 49)

##### Work emotionally

Although most of the care staff were equipped with limited knowledge of dementia, an overall positive attitude towards taking care of residents with dementia was observed, as noted by one participant:

*‘Seeing them improve their physical condition through my care, I truly feel satisfied. I am accumulating good deeds and this work is meaningful to me.’* (Yang et al., 2024, p. 104)

Even though, some participants argued that good manners, kindness, caring, patience or humour in caregiving interactions with people with dementia in daily care play a greater role than knowledge of brain pathology or formal professional education (Strom et al., 2021; van Wyk et al., 2017; Wang et al., 2022; Yektatalab et al., 2012). From the study carried out by Siewert et al. (2021), one of the reasons underlying the effectiveness of ‘emotional care’ is the emotional engagement of caregivers, which becomes a powerful tool for building bonds with people with dementia.

Some participants, however, also shared their emotional burdens, such as guilt, anxiety and burnout, due to their lack of capacity to provide care for people with dementia effectively (Md Hussin et al., 2023; Yang et al., 2024). In addition, the characteristics of participants, such as religious and cultural beliefs and embodied empathy and sympathy for people with dementia, worked as intrinsic motivators and contributed to a compassionate caregiving experience for caregivers (Jiang et al., 2022; Strom et al., 2021; Yang et al., 2024). Participants in one study shared that they preferred to recruit care staff who believe in God because they tended to be more patient (Jiang et al., 2022), as one participant noted:

*‘We organize employees to pray... (We use religion to improve employees’ empathy). When we see older adults, we think of ourselves because we will get old… (and) we think of our parents.’ *(Jiang et al., 2022, p. 781)

##### Group activities

Group activities provided for residents with dementia were valued as important means of socio-cultural care, promoting an inclusive community. The interpersonal interaction integrated into the group activities has been considered an efficient strategy for addressing distressed behaviours among residents with dementia (van Wyk et al., 2017). However, from the literature synthesis, no structured and standardised group activities were arranged among the care home settings; the group activities provided included religious praying activities (Yektatalab et al., 2012), student volunteer visits (Jiang et al., 2022), and watching movies (Zhao et al., 2021). This may indicate a need for attention to developing widely applicable group activities for residents with dementia within the LTC settings.

#### The deficiencies in caregiving approaches that contributed to poor-quality care for people living with dementia

##### Care practices that overlook the self-care ability of people with dementia

There was a phenomenon of care practices that overlooked the self-care ability of people with dementia observed in the literature. For example, a care practice involving a care assistant helping a resident with dementia to use the towel; however, this resident still could do this by herself, and she thought that the movement of that resident was too sluggish, but she could help to do it more efficiently (Zhao et al., 2021). Similar care situations that ignored the independence of people with dementia also happened in some daily care activities, such as feeding and bathing, and the potential causes of these were regarded as the inadequate time of care staff to provide care, influenced by the task-oriented care (Wang et al., 2022) and the facility’s busy work routine (Siewert et al., 2021). It also points to a lack of training and understanding that helping someone too much can disempower them, but it’s a training issue, and the carers couldn’t be expected to navigate that without appropriate training.

##### Unmet needs of people with dementia during caregiving

From the illustration of the study conducted by Wang et al. (2022), there was a tendency for people with dementia with impaired communication ability to face the risk of being ignored and having unmet needs during caregiving, especially for advanced dementia or those who were completely bedridden. de Melo et al. (2023) pointed out that caregivers encountered major difficulty during the pandemic due to the conflicts between addressing the needs of people with dementia and the restrictions raised due to COVID-19. Such a situation worsens because of the cognitive impairment of people with dementia and leaves caregivers feeling unable to communicate effectively with them, as described in the following quotes:

*‘It [COVID-19] impacted the care format due to the social distance, for not understanding the circumstances, the PLWD became stressed and anxious making an impact in patient care.’ *(de Melo et al., 2023, p. 48)

As well as the communication difficulties caused by the functional ability impairment of people with dementia, caregivers also expressed their concerns about their inability to comprehend and respond to behavioural messages of people with dementia, which leaves their needs unmet (Wang et al., 2022; Yektatalab et al., 2012). Besides, the facility’s busy routine directly affected the time caregivers had to provide care and correspondingly influenced the quality of care. For instance, caregivers stated their worries concerning complying with the facility’s schedules and reducing adequate engagement to complete tasks (Siewert et al., 2021).

*‘Sometimes, you’d give a better bath, not that we provide a poor quality bath, but you know, time runs. It’s our routine; there’s nothing we can do about.’* (Siewert et al., 2021, p. 8)

##### Need to provide care with dignity

As the dominant aspect of quality care for people living with dementia, the provision of dignified care and the preservation of autonomy and personhood for people with dementia were found to be inadequately fulfilled. This was influenced by the limited knowledge and inappropriate understanding of dementia of caregivers. This becomes evident through the rigidity of care routines and activity arrangements within LTC settings, which are standardised to accommodate the staff’s working schedules but often lack consideration and flexibility concerning residents’ autonomy in selecting preferred care services (Siewert et al., 2021; Zhao et al., 2021), as one participant stated:

*‘They have lunchtime, sleep time, coffee time, and a time they go to mass. We have to work within this schedule.’ *(Siewert et al., 2021, p. 8)

The changes related to COVID-19 precautions impacted the autonomy of residents with dementia as well, such as the physical distancing didn’t allow them to walk around with autonomy (de Melo et al., 2023). In addition, the imbalanced power relationship between residents with dementia and their caregivers did not protect their dignity within the care interaction. This was reflected in the authority of caregivers and the patronising communication style observed in their daily care (Wang et al., 2022; Zhao et al., 2021). For instance:

*‘When communicating with them (residents with dementia), we tried to pamper them like coaxing children by flattery. Older adults were just like children. For example, say “follow my order and I’ll get you some tasty food”; “very good, just keep on like that, good boy”.’* (Zhao et al., 2021, p. 6)

##### Inadequate management of the changed behaviours of people with dementia

Taking care of people living with dementia who present changed behaviours, also referred to as behavioural and psychological symptoms of dementia (BPSD), is always challenging for the caregivers, and these unmanaged changed behaviours significantly consume the caregivers’ attention and time during dementia care practices. In addition to employing coping strategies aimed at distracting and calming residents with dementia who are restless (Md Hussin et al., 2023; Zhao et al., 2021), caregivers tended to rely on sedative medication as an effective means of managing BPSD (van Wyk et al., 2017), such as experiences from participants:

*‘Sometimes they are so overmedicated and are like “zombies”. It is not nice if they are like that, because you cannot work with them if they are in that state.’* (van Wyk et al., 2017, p. 7)

Concurrently, the reliance on medication was attributed to the perceived lack of alternative viable options to address such challenging behaviours adequately. Moreover, a disconcerting aspect is the prevalent adoption of physical restraint and isolation as strategies to manage behavioural challenges exhibited by residents with dementia in a state of agitation (Md Hussin et al., 2023; van Wyk et al., 2017; Zhao et al., 2021), as quoted below:

*‘Sometimes you have to restrain them until the medication calms them down.’ *(van Wyk et al., 2017, p. 7)

*‘We just have to leave them alone for a while, let them release the anger and agitation inside them, then only we talk to them again. We cannot totally ignore them …. However, it’s very important to give time to them.’* (Md Hussin et al., 2023, p. 499)

These measures raise ethical concerns due to the potential detriment to the well-being and dignity of individuals with dementia and underscore how significantly education and training deficits impact care.

## Discussion

The synthesised findings provided novel insights into the care experience of LTC staff of people living with dementia in the context of LMICs. Overall, underdeveloped dementia care services among LTC settings were demonstrated, and three categories were suggested as associated with the participants’ positive and negative experiences during their caregiving process.

The development of dementia care within care home settings has an important relationship with the contextual factors of social, political and economic conditions. In our review, the issue of the absence of a national dementia strategy in Nepal (Shrestha & Tranvag, 2022) and South Africa (van Wyk et al., 2017) was raised by researchers, who valued the idea of a national dementia strategy as necessary for promoting dementia care. Regrettably, national dementia plans, an important initiative of the World Health Organization (WHO) to address the dementia challenge, have not yet been fully implemented and require further attention in LMICs (World Health Organization, 2017, 2021). It was common that the lack of sufficient financial funding from the government resulted in the struggling operation of the care home settings; even more, the cumbersome administrative procedures and regional disparity were affecting the development of dementia care services in care home settings (Jiang et al., 2022; Shrestha & Tranvag, 2022). Thus, adequate policy responses to dementia care from the governmental level should make dementia care a priority for healthcare providers and local authorities through the formulation of national dementia plans and the provision of sufficient funding to support its development.

The understaffing of the care staff workforce and the resulting busy daily routines were considered factors that restricted the quality of dementia care. For instance, participants shared experiences indicating that they had to rush into complying with the scheduled daily routines without regard to the quality of care (Wang et al., 2022). To some extent, the communication between caregivers and people with dementia was challenging due to heavy workloads; participants shared that they did not have much time to talk with the residents when taking care of them (Yektatalab et al., 2012). A similar perception was shared by van Wyk et al. (2017) and Md Hussin et al. (2023), participants indicated that more staff are needed so they can spend more time caring for older people living with dementia, particularly those who were experiencing changed behaviours. Maintaining adequate staffing levels in LTC facilities is crucial for improving care quality (Gilster et al., 2018). This has been identified as a basic need that must be addressed in LTC settings, particularly within the resource-constrained contexts of LMICs.

The issue of understaffing in LTC facilities in LMICs was evident not only in the shortage of general care staff but also in the limited involvement of professional health workers. In many cases, only basic daily care services were available, while key roles such as physiotherapists, activity facilitators, and social workers were largely absent from dementia care practices in these settings. The World Alzheimer Report 2022 (Gauthier et al., 2022) advocates for establishing multidisciplinary teams to assist people living with dementia in navigating the care pathway and receiving optimal care services at each stage of the dementia journey. One example of such a care service is cognitive stimulation, a structured approach to engaging people with dementia in mentally stimulating activities that can help maintain cognitive function. Woods et al. (2012) conducted a systematic review that suggested the beneficial effect of cognitive stimulation on memory and thinking test scores and improved quality of life of people with dementia. Thus, a well-rounded care team composed of professionals from multiple disciplines is needed to facilitate comprehensive care services for people with dementia living in LTC settings.

Based on the nature of teamwork in the caregiving process, team collaboration and peer support were acknowledged and valued as pivotal sources of fostering nursing and care skills development by the participants, particularly in coping with the changed behaviours of dementia. For example, caregivers actively learned useful strategies from their colleagues, and they also collaborated by sharing the nursing difficulties they faced and coming up with appropriate coping strategies that could address those challenges (Siewert et al., 2021). At the same time, participants acknowledged managers’ leadership as an important facilitator of their sense of self-competence and achievement. They regarded the managers as ‘role models’, consistent with the positive impact of leaders’ supervision, support, and encouragement (Jiang et al., 2022; van Wyk et al., 2017; Yang et al., 2024). The impact of leadership, such as promoting a healthy and supportive work environment, was recognised as important to ensure and safeguard staff health and well-being, along with the maintenance of the quality of care (Backman et al., 2023).

The skills and competencies of carers are critical to the quality of dementia care they deliver. To support them in this demanding role, it is essential to offer adequate resources, practical training, and educational preparation that enhance their understanding of dementia and their ability to provide effective care. Studies included in this systematic review showed a common characteristic of the participants whom with overall limited knowledge and skills in delivering dementia care. The dearth of dementia care knowledge among care staff created a feeling of insufficient preparation and reduced confidence in providing appropriate dementia care. That was made worse by the unavailability of training resources and ineffective training for LTC staff in LMICs (Seeher et al., 2023). Most of the current training was not standard, and the content primarily covered basic daily care skills, with less emphasis on dementia-specific care. Thus, it was insufficient for the capacity building of caregivers to provide quality dementia care (Md Hussin et al., 2023; Shrestha & Tranvag, 2022; Yang et al., 2024; Yektatalab et al., 2012). In addition, the participants raised the lack of tailored training content to caregivers’ education levels (Wang et al., 2022). This is particularly important where caregivers lack formal basic education or have limited literacy, which impedes access to resources. The above deficiency in available training left participants with negative and questioning attitudes towards the training (Wang et al., 2022).

Despite poorly prepared dementia care training, this review also discovered an inspiring phenomenon of the participants’ positive attitude towards training and education in dementia care. Care staff valued the training that emphasised practical skills, expressing enthusiasm towards such training as an important means of enhancing care competencies, particularly for facilitating their ability to manage the changed behaviours of people with dementia (Md Hussin et al., 2023; Shrestha & Tranvag, 2022; Zhao et al., 2021).

Most care staff agreed that training tailored to dementia care would significantly enhance their capacity and boost their confidence in providing care for residents with dementia (Md Hussin et al., 2023; van Wyk et al., 2017). Within this particular context, there is a pressing necessity to formulate and develop a culturally adapted training programme on dementia care for the LTC staff in LMICs.

## Strengths and limitations

The strength of this review lies in the rigorous approach to the comprehensive literature search to identify the eligible studies. To guarantee adequate and efficient coverage of the literature searching, the databases we searched were combined with the commonly used sources of medical and nursing relevant disciplines. The decision to feature studies from the context of LMICs could be regarded as an advantage, as the development of dementia care in under-resourced areas may need the corresponding and unique pathway based on the understanding of certain contexts, and that is what our findings hope to provide.

Additional methodological limitations should be acknowledged. While the included studies were of acceptable quality according to the CASP checklist (all scoring 16 or more out of 20), a consistent limitation across studies was inadequate consideration of researcher-participant relationships, indicating limited transparency around how researchers’ assumptions, positions, or cultural contexts influenced data collection and interpretation. This is particularly important in cross-cultural contexts where power dynamics between researchers and care staff may affect the authenticity of reported experiences. Further research should employ more rigorous reflexivity practices to acknowledge and address the researcher’s positionality in relation to participants.

Besides, only studies published in English were included, which represents a notable limitation given that most LMICs are non-English speaking countries. This restriction may have introduced language bias by excluding potentially relevant research published in local languages, particularly studies that might capture unique cultural nuances in dementia care approaches. While English remains the predominant language for international academic publication, this limitation potentially affects the comprehensiveness of our findings, especially for regions where research is frequently published in languages such as Mandarin, Spanish, Portuguese, or Arabic. Future reviews would benefit from incorporating multilingual search capabilities and fostering international collaboration to enhance the inclusivity and comprehensiveness of synthesised qualitative evidence.

Additionally, the studies were drawn from a relatively small number of LMICs, meaning that regional representation was uneven, and the synthesis may not capture the full diversity of care contexts. Crucially, future studies should seek broader geographic representation within LMICs to ensure the cultural relevance and applicability of findings across diverse contexts. Moreover, incorporating the perspectives of people living with dementia and their families as co-participants in research is recommended to ensure interventions are truly responsive to the lived realities and needs of those directly affected by dementia care policies and practices. Besides, the absence of qualitative research with participants in LTC settings from LMICs may imply that the findings represent an underestimation of the true reality.

## Recommendations for practice and policy

This review highlights the urgent need to enhance the dementia care competence of LTC staff in LMICs. Training programmes should not only address essential care strategies such as managing behavioural symptoms and communication but also be culturally adapted to caregiving values and expectations unique to each setting. In societies where filial responsibility is deeply embedded (e.g., in many Asian and Middle Eastern cultures), training should include strategies for engaging families, negotiating shared care responsibilities, and addressing interpersonal dynamics around institutionalisation. Additionally, where stigma and misinformation about dementia persist, staff education should incorporate anti-stigma training and culturally sensitive communication.

Resource-adapted environmental modifications represent another crucial area for culturally sensitive intervention. In LMICs where purpose-built dementia care environments may be financially unfeasible, low-cost adaptations that reflect familiar cultural elements should be prioritised. Such approaches acknowledge that effective dementia-friendly environments need not follow Western institutional models but can instead build upon existing cultural strengths and spatial practices unique to each region.

Meanwhile, it is also recommended that the development and support scheme for caregivers should cater to the adaptation of long-term residential dementia care in certain social, cultural, and political environments. Policymakers in LMICs should prioritise the development of context-specific dementia care frameworks that address the needs of both care recipients and staff. Investment in training and support systems should align with local cultural expectations and healthcare infrastructure, which will be key to improving care quality.

## Conclusion

In this qualitative systematic review, we reviewed the studies that explored the care experience of LTC staff of people with dementia in LMICs. Currently, there is limited support available for building dementia care competencies of LTC staff to provide quality care to people living with dementia within the context of LMICs. Although our findings show that the development of dementia care within LTC settings in LMICs is in its infancy and not encouraging, uplifting positive coping strategies were proven from this systematic review, and such positive experiences can evoke insight into the further development of dementia care services in LTC settings in LMICs. We hope this review will draw greater attention to the critical and underdeveloped area of long-term institutional care for people with dementia in LMICs and inspire further research on this topic.

## Supplemental Material

Supplemental Material - The experience of long-term care staff caring for people with dementia in low- and middle-income Countries (LMICs): A qualitative evidence synthesis Supplemental Material for The experience of long-term care staff caring for people with dementia in low- and middle-income Countries (LMICs): A qualitative evidence synthesis by Xiaoyang Li, Heather Wilkinson, Mengying Zhang, and Sarah J Rhynas in Dementia.

Supplemental Material - The experience of long-term care staff caring for people with dementia in low- and middle-income Countries (LMICs): A qualitative evidence synthesis Supplemental Material for The experience of long-term care staff caring for people with dementia in low- and middle-income Countries (LMICs): A qualitative evidence synthesis by Xiaoyang Li, Heather Wilkinson, Mengying Zhang, and Sarah J Rhynas in Dementia.

Supplemental Material - The experience of long-term care staff caring for people with dementia in low- and middle-income Countries (LMICs): A qualitative evidence synthesis Supplemental Material for The experience of long-term care staff caring for people with dementia in low- and middle-income Countries (LMICs): A qualitative evidence synthesis by Xiaoyang Li, Heather Wilkinson, Mengying Zhang, and Sarah J Rhynas in Dementia.
