# Supplementary material for: The experience of long-term care staff caring for people with dementia in low- and middle-income Countries (LMICs): A qualitative evidence synthesis
Source: Dementia (London). 2025 May 28;25(1):173–91. doi: 10.1177/14713012251346597 (PMC12701081; doi:10.1177/14713012251346597)
Supplement: Supplemental Material - The experience of long-term care staff caring for people with dementia in low- and middle-income Countries (LMICs): A qualitative evidence synthesis [file sj-pdf-1-dem-10.1177_14713012251346597.pdf]

Supplementary materials: The experience of long-term care staff caring for people with dementia in low- and middle-income Countries (LMICs): A qualitative evidence synthesis

Title: Appendix 1: Search Strategies
Description: This appendix provides a detailed account of the search strategies used to identify relevant studies for inclusion in the review. It outlines the databases searched, the search terms and keywords applied, and any filters or limits set during the search process.

Title: Appendix 2: Characteristics of the Included Studies
Description: This appendix presents an overview of the key characteristics of the studies included in the review. It includes information such as author, year, country, study objectives, study design, contributing participants, care settings and key findings.

Title: Appendix 3: CASP Quality Appraisal Scoring Tables
Description: This appendix contains the results of the quality appraisal conducted using the Critical Appraisal Skills Programme (CASP) tool. Each included study is evaluated based on the CASP criteria, and the results are presented in a scoring table format. The table highlights the strengths and limitations of each study, providing a transparent assessment of their methodological rigour.

What is the long-term care staff's experience of caring for people living with dementia in low- and middle-income Countries (LMICs)? A qualitative evidence synthesis

Table 1. The SPIDER tool applied to the review question

Sample (S)	Nursing home staff of people with dementia in Low- and Middle- Income country
Phenomena of Interest (PI)	Long-term care staff who care for people with dementia
Design (S)	interview, focus group, observation, discussion (qualitative research design)
Evaluation (E)	Studies related to the experiences of long-term care staff of people with dementia
Research type (R)	qualitative studies and mixed-method studies with a qualitative component

Table 2. Search terms mapped against the SPIDER tool

	Subject terms/heading	***keywords***
Sample (S)	***Subject terms/heading:*** Dementia Alzheimer's Disease Dementia, Vascular	dement* OR alzheimer* OR "alzheimer disease" OR AD OR "vascular dementia" OR "lewy body disease" OR "frontotemporal dementia" OR "frontotemporal lobar degeneration" OR "multi infarct dementia" OR "multi-infarct dementia" OR "mixed dementia"

	Lewy Body Disease<br>Frontotemporal lobar degeneration<br>Multi infarct dementia<br><br>***Subject terms/heading:***<br>Nursing home personnel<br>Caregiver<br>Nurse<br>Nursing assistant<br>Health personnel	"nursing home personnel*" OR "nursing home employee*" OR "nursing home staff*" OR "nursing home care staff*" OR "nursing home assistant*" OR "nursing home caregiv* staff*" OR "care home staff*" OR caregiv* OR carer* OR "care giv*" OR "care staff*" OR careworker* OR "care worker*" OR nurs* OR "registered nurse*" OR RN OR "licen?ed practical nurse*" OR LPN OR "nurs* staff*" OR "nurs* aid*" OR "care aid*" OR "nurs* assistant*" OR "care attendant*" OR "nurs* auxilliar*" OR "support worker*" OR "healthcare worker*" OR "healthcare assistant*" OR "healthcare staff*" OR "health personnel*" OR "health care personnel*" OR "health care assistant*" OR "healthcare professional*" OR staff* OR "support staff*" OR worker* OR employee* OR practitioner* OR personnel* OR manager* OR leader*
Phenomena of Interest (PI)	***Subject terms/heading:***<br>Nursing home<br>Residential home<br>Residential facilities<br>Institutional care	"nursing home*" OR "nursing facilit*" OR "skilled nursing facilit*" OR "resident* facilit*" OR "resident* home*" OR "care home*" OR "long term care" OR "long-term care" OR "long term care setting" OR "long term care institution*" OR "long term care facilit*" OR "long-term care setting*" OR "long-term care institution*" OR "long-term facilit*" OR "resident* care institution*" OR "care facilit*" OR "health care facilit*" OR "aged care facilit*" OR "institut* care" OR "institut* care setting*" OR "institut* care facilit*"
Design (D)	***Subject terms/heading:***<br>Interview as topic<br>Semi-structured interview<br>Structured	((semi-structured or semistructured or unstructured or informal or in-depth or indepth or "face to face" or face-to-face or structured or guide) and (interview* or discussion* or questionnaire*)) or "focus group*" or observ* or fieldwork or "field work" or "key informant"

	interview<br>Group discussion	
Evaluation (E)		((semi-structured or semistructured or unstructured or informal or in-depth or indepth or "face to face" or face-to-face or structured or guide) and (interview* or discussion* or questionnaire*)) or "focus group*" or observ* or fieldwork or "field work" or "key informant"
Research type (R)	***Subject terms/heading:***<br>Qualitative research<br>Qualitative studies	"qualitative research" or "qualitative study" or qualitative or narrat* or ethnograph* or phenomeno* or IPA or "interpretative phenomenological analysis" or "grounded theory" or hermeneutic* or "mixed method*" or mixed-method* or "multi method*" or multi-method*

Search details in each database
Table 3. CINAHIL

#	Query
S1	(MH "Dementia+") OR (MH "Frontotemporal Dementia+") OR (MH "Dementia, Vascular+") OR (MH "Dementia, Multi-Infarct") OR (MH "Lewy Body Disease")
S2	(MH "Alzheimer's Disease")
S3	TX dement* OR alzheimer* OR "alzheimer disease" OR AD OR "vascular dementia" OR "lewy body disease" OR "frontotemporal dementia" OR "frontotemporal lobar degeneration" OR "multi infarct dementia" OR "multi-infarct dementia" OR "mixed dementia"
S4	S1 OR S2 OR S3
S5	(MH "Nursing Home Personnel")
S6	(MH "Caregivers")
S7	(MH "Nurses+") OR (MH "Nursing Assistants")
S8	(MH "Health Personnel+")
S9	TX "nursing home personnel*" OR "nursing home employee*" OR "nursing home staff*" OR "nursing home care staff*" OR "nursing home assistant*" OR "nursing home caregiv* staff*" OR "care home staff*" OR caregiv* OR carer* OR "care giv*" OR "care staff*" OR careworker* OR "care worker*" OR nurs* OR "registered nurse*" OR RN OR "licen?ed practical nurse*" OR LPN OR "nurs* staff*" OR "nurs* aid*" OR "care aid*" OR "nurs* assistant*" OR "care attendant*" OR "nurs* auxilliar*" OR "support worker*" OR "healthcare worker*" OR "healthcare assistant*" OR "healthcare staff*" OR "health personnel*" OR "health care personnel*" OR "health care assistant*" OR "healthcare professional*" OR staff* OR "support staff*" OR worker* OR employee* OR

	practitioner* OR personnel* OR manager* OR leader*
S10	S5 OR S6 OR S7 OR S8 OR S9
S11	(MH "Nursing Homes+") OR (MH "Skilled Nursing Facilities") OR (MH "Residential Facilities+")
S12	TX "nursing home*" OR "nursing facilit*" OR "skilled nursing facilit*" OR "resident* facilit*" OR "resident* home*" OR "care home*" OR "long term care" OR "long-term care" OR "long term care setting" OR "long term care institution*" OR "long term care facilit*" OR "long-term care setting*" OR "long-term care institution*" OR "long-term facilit*" OR "resident* care institution*" OR "care facilit*" OR "health care facilit*" OR "aged care facilit*" OR "institut* care" OR "institut* care setting*" OR "institut* care facilit*"
S13	S11 OR S12
S14	TX experience* OR encounter* OR perspect* OR percept* OR opinion* OR attitud* OR view* OR viewpoint* OR standpoint* OR feel* OR belief* OR thought* OR support* OR need* OR requir* OR demand* OR expect* OR wish*
S15	(MH "Interviews+") OR (MH "Semi-Structured Interview") OR (MH "Structured Interview")
S16	(MH "Focus Groups")
S17	(MH "Qualitative Studies+")
S18	TI (((semi-structured OR semistructured OR unstructured OR informal OR in-depth OR indepth OR “face to face” OR face-to-face OR structured OR guide) AND (interview* OR discussion* OR questionnaire*)) OR (“focus group*” OR observ* OR fieldwork OR “field work” OR “key informant”)) OR AB (((semi-structured OR semistructured OR unstructured OR informal OR in-depth OR indepth OR “face to face” OR face-to-face OR structured OR guide) AND (interview* OR discussion* OR questionnaire*)) OR (“focus group*” OR observ* OR fieldwork OR “field work” OR “key informant”))
S19	TI ("qualitative research" OR "qualitative study" OR qualitative OR narrat* OR ethnograph* OR phenomeno* OR IPA OR "interpretative phenomenological analysis" OR "grounded theory" OR hermeneutic* OR "mixed method*" OR mixed-method* OR "multi method*" OR multi-method*) OR AB (qualitative research" OR "qualitative study" OR qualitative OR narrat* OR ethnograph* OR phenomeno* OR IPA OR "interpretative phenomenological analysis" OR "grounded theory" OR hermeneutic* OR "mixed method*" OR mixed-method* OR "multi method*” OR multi-method*)
S20	S15 OR S16 OR S17 OR S18 OR S19
S21	S4 AND S11 AND AND S13 AND S14 AND S20 (Narrow by Language: -English)

Table 4. EMBASE

#	**Query**
1	exp multiinfarct dementia/ or exp dementia/ or exp frontotemporal dementia/
2	exp Alzheimer disease/
3	exp diffuse Lewy body disease/
4	(dement* or alzheimer* or "alzheimer disease" or AD or "vascular dementia" or "lewy body disease" or "frontotemporal dementia" or "frontotemporal lobar degeneration"

	or "multi infarct dementia" or "multi-infarct dementia" or "mixed dementia").mp. [mp=title, abstract, heading word, drug trade name, original title, device manufacturer, drug manufacturer, device trade name, keyword heading word, floating subheading word, candidate term word]
5	1 or 2 or 3 or 4
6	nursing home personnel/
7	exp caregiver/
8	exp nurse/
9	exp nursing assistant/
10	exp health care personnel/
11	("nursing home personnel*" or "nursing home employee*" or "nursing home staff*" or "nursing home care staff*" or "nursing home assistant*" or "nursing home caregiv* staff*" or "care home staff*" or caregiv* or carer* or "care giv*" or "care staff*" or careworker* or "care worker*" or nurs* or "registered nurse*" or RN or "licen?ed practical nurse*" or LPN or "nurs* staff*" or "nurs* aid*" or "care aid*" or "nurs* assistant*" or "care attendant*" or "nurs* auxilliar*" or "support worker*" or "healthcare worker*" or "healthcare assistant*" or "healthcare staff*" or "health personnel*" or "health care personnel*" or "health care assistant*" or "healthcare professional*" or staff* or "support staff*" or worker* or employee* or practitioner* or personnel* or manager* or leader*).mp. [mp=title, abstract, heading word, drug trade name, original title, device manufacturer, drug manufacturer, device trade name, keyword heading word, floating subheading word, candidate term word]
12	6 or 7 or 8 or 9 or 10 or 11
13	exp nursing home/
14	exp residential home/
15	exp institutional care/
16	("nursing home*" or "nursing facilit*" or "skilled nursing facilit*" or "resident* facilit*" or "resident* home*" or "care home*" or "long term care" or "long-term care" or "long term care setting" or "long term care institution*" or "long term care facilit*" or "long-term care setting*" or "long-term care institution*" or "long-term facilit*" or "resident* care institution*" or "care facilit*" or "health care facilit*" or "aged care facilit*" or "institut* care" or "institut* care setting*" or "institut* care facilit*").mp. [mp=title, abstract, heading word, drug trade name, original title, device manufacturer, drug manufacturer, device trade name, keyword heading word, floating subheading word, candidate term word]
17	13 or 14 or 15 or 16
18	(experience* or encounter* or perspect* or percept* or opinion* or attitud* or view* or viewpoint* or standpoint* or feel* or belief* or thought* or support* or need* or requir* or demand* or expect* or wish*).mp. [mp=title, abstract, heading word, drug trade name, original title, device manufacturer, drug manufacturer, device trade name, keyword heading word, floating subheading word, candidate term word]
19	exp interview/ or exp structured interview/ or exp semi structured interview/
20	exp qualitative research/

21	(((semi-structured or semistructured or unstructured or informal or in-depth or indepth or "face to face" or face-to-face or structured or guide) and (interview* or discussion* or questionnaire*)) or "focus group*" or observ* or fieldwork or "field work" or "key informant").ab,ti.
22	("qualitative research" or "qualitative study" or qualitative or narrat* or ethnograph* or phenomeno* or IPA or "interpretative phenomenological analysis" or "grounded theory" or hermeneutic* or "mixed method*" or mixed-method* or "multi method*" or multi-method*).ab,ti.
23	19 or 20 or 21 or 22
24	5 and 12 and 17 and 18 and 23
25	limit 24 to english language

Table 5. MEDLINE

#	Query
1	exp Dementia/ or exp Frontotemporal Dementia/ or exp Dementia, Vascular/ or exp Dementia, Multi-Infarct/
2	exp Alzheimer Disease/
3	exp Lewy Body Disease/
4	(dement* or alzheimer* or "alzheimer disease" or AD or "vascular dementia" or "lewy body disease" or "frontotemporal dementia" or "frontotemporal lobar degeneration" or "multi infarct dementia" or "multi-infarct dementia" or "mixed dementia").mp. [mp=title, book title, abstract, original title, name of substance word, subject heading word, floating sub-heading word, keyword heading word, organism supplementary concept word, protocol supplementary concept word, rare disease supplementary concept word, unique identifier, synonyms]
5	1 or 2 or 3 or 4
6	exp Caregivers/
7	exp Nurses/
8	exp Nursing Assistants/
9	exp Health Personnel/
10	("nursing home personnel*" or "nursing home employee*" or "nursing home staff*" or "nursing home care staff*" or "nursing home assistant*" or "nursing home caregiv* staff*" or "care home staff*" or caregiv* or carer* or "care giv*" or "care staff*" or careworker* or "care worker*" or nurs* or "registered nurse*" or RN or "licen?ed practical nurse*" or LPN or "nurs* staff*" or "nurs* aid*" or "care aid*" or "nurs* assistant*" or "care attendant*" or "nurs* auxilliar*" or "support worker*" or "healthcare worker*" or "healthcare assistant*" or "healthcare staff*" or "health personnel*" or "health care personnel*" or "health care assistant*" or "healthcare professional*" or staff* or "support staff*" or worker* or employee* or practitioner* or personnel* or manager* or leader*).mp. [mp=title, book title, abstract, original title, name of substance word, subject heading word, floating sub-heading word, keyword heading word, organism supplementary concept word, protocol supplementary concept word, rare disease supplementary concept word, unique

	identifier, synonyms]
11	6 or 7 or 8 or 9 or 10
12	exp Residential Facilities/
13	exp Assisted Living Facilities/
14	exp Nursing Homes/
15	("nursing home*" or "nursing facilit*" or "skilled nursing facilit*" or "resident* facilit*" or "resident* home*" or "care home*" or "long term care" or "long-term care" or "long term care setting" or "long term care institution*" or "long term care facilit*" or "long-term care setting*" or "long-term care institution*" or "long-term facilit*" or "resident* care institution*" or "care facilit*" or "health care facilit*" or "aged care facilit*" or "institut* care" or "institut* care setting*" or "institut* care facilit*").mp. [mp=title, book title, abstract, original title, name of substance word, subject heading word, floating sub-heading word, keyword heading word, organism supplementary concept word, protocol supplementary concept word, rare disease supplementary concept word, unique identifier, synonyms]
16	12 or 13 or 15
17	(experience* or encounter* or perspect* or percept* or opinion* or attitud* or view* or viewpoint* or standpoint* or feel* or belief* or thought* or support* or need* or requir* or demand* or expect* or wish*).mp. [mp=title, book title, abstract, original title, name of substance word, subject heading word, floating sub-heading word, keyword heading word, organism supplementary concept word, protocol supplementary concept word, rare disease supplementary concept word, unique identifier, synonyms]
18	exp Qualitative Research/
19	exp Interview/
20	exp Focus Groups/
21	(((semi-structured or semistructured or unstructured or informal or in-depth or indepth or "face to face" or face-to-face or structured or guide) and (interview* or discussion* or questionnaire*)) or "focus group*" or observ* or fieldwork or "field work" or "key informant").ab,ti.
22	("qualitative research" or "qualitative study" or qualitative or narrat* or ethnograph* or phenomeno* or IPA or "interpretative phenomenological analysis" or "grounded theory" or hermeneutic* or "mixed method*" or mixed-method* or "multi method*" or multi-method*).ab,ti.
23	18 or 19 or 20 or 21 or 22
24	5 and 11 and 16 and 17 and 23
25	limit 24 to english language

Table6. PsycINFO

#	Query
1	exp Dementia/ or exp Dementia with Lewy Bodies/ or exp Vascular Dementia/
2	exp Alzheimer's Disease/

3	(dement* or alzheimer* or "alzheimer disease" or AD or "vascular dementia" or "lewy body disease" or "frontotemporal dementia" or "frontotemporal lobar degeneration" or "multi infarct dementia" or "multi-infarct dementia" or "mixed dementia").mp. [mp=title, abstract, heading word, table of contents, key concepts, original title, tests & measures, mesh word]
4	1 or 2 or 3
5	exp health personnel/ or exp allied health personnel/ or exp caregivers/
6	exp Nurses/
7	("nursing home personnel*" or "nursing home employee*" or "nursing home staff*" or "nursing home care staff*" or "nursing home assistant*" or "nursing home caregiv* staff*" or "care home staff*" or caregiv* or carer* or "care giv*" or "care staff*" or careworker* or "care worker*" or nurs* or "registered nurse*" or RN or "licen?ed practical nurse*" or LPN or "nurs* staff*" or "nurs* aid*" or "care aid*" or "nurs* assistant*" or "care attendant*" or "nurs* auxilliar*" or "support worker*" or "healthcare worker*" or "healthcare assistant*" or "healthcare staff*" or "health personnel*" or "health care personnel*" or "health care assistant*" or "healthcare professional*" or staff* or "support staff*" or worker* or employee* or practitioner* or personnel* or manager* or leader*).mp. [mp=title, abstract, heading word, table of contents, key concepts, original title, tests & measures, mesh word]
8	5 or 6 or 7
9	exp nursing homes/ or exp residential care institutions/
10	("nursing home*" or "nursing facilit*" or "skilled nursing facilit*" or "resident* facilit*" or "resident* home*" or "care home*" or "long term care" or "long-term care" or "long term care setting" or "long term care institution*" or "long term care facilit*" or "long-term care setting*" or "long-term care institution*" or "long-term facilit*" or "resident* care institution*" or "care facilit*" or "health care facilit*" or "aged care facilit*" or "institut* care" or "institut* care setting*" or "institut* care facilit*").mp. [mp=title, abstract, heading word, table of contents, key concepts, original title, tests & measures, mesh word]
11	9 or 10
12	(experience* or encounter* or perspect* or percept* or opinion* or attitud* or view* or viewpoint* or standpoint* or feel* or belief* or thought* or support* or need* or requir* or demand* or expect* or wish*).mp. [mp=title, abstract, heading word, table of contents, key concepts, original title, tests & measures, mesh word]
13	exp Qualitative Methods/
14	exp interviews/ or exp focus group interview/ or exp semi-structured interview/
15	(((semi-structured or semistructured or unstructured or informal or in-depth or indepth or "face to face" or face-to-face or structured or guide) and (interview* or discussion* or questionnaire*)) or "focus group*" or observ* or fieldwork or "field work" or "key informant").ab,ti.
16	("qualitative research" or "qualitative study" or qualitative or narrat* or ethnograph* or phenomeno* or IPA or "interpretative phenomenological analysis" or "grounded theory" or hermeneutic* or "mixed method*" or mixed-method* or "multi method*"

	or multi-method*).ab,ti.
17	13 or 14 or 15 or 16
18	4 and 8 and 11 and 12 and 17
19	limit 18 to english language

Table7. ASSIA

Set#	Searched for
S1	noft(dement* OR alzheimer* OR "alzheimer disease" OR AD OR "vascular dementia" OR "lewy body disease" OR "frontotemporal dementia" OR "frontotemporal lobar degeneration" OR "multi infarct dementia" OR "multi-infarct dementia" OR "mixed dementia")
S2	noft("nursing home personnel*" OR "nursing home employee*" OR "nursing home staff*" OR "nursing home care staff*" OR "nursing home assistant*" OR "nursing home caregiv* staff*" OR "care home staff*" OR caregiv* OR carer* OR "care giv*" OR "care staff*" OR careworker* OR "care worker*" OR nurs* OR "registered nurse*" OR RN OR "licen?ed practical nurse*" OR LPN OR "nurs* staff*" OR "nurs* aid*" OR "care aid*" OR "nurs* assistant*" OR "care attendant*" OR "nurs* auxilliar*" OR "support worker*" OR "healthcare worker*" OR "healthcare assistant*" OR "healthcare staff*" OR "health personnel*" OR "health care personnel*" OR "health care assistant*" OR "healthcare professional*" OR staff* OR "support staff*" OR worker* OR employee* OR practitioner* OR personnel* OR manager* OR leader*)
S3	noft("nursing home*" OR "nursing facilit*" OR "skilled nursing facilit*" OR "resident* facilit*" OR "resident* home*" OR "care home*" OR "long term care" OR "long-term care" OR "long term care setting" OR "long term care institution*" OR "long term care facilit*" OR "long-term care setting*" OR "long-term care institution*" OR "long-term facilit*" OR "resident* care institution*" OR "care facilit*" OR "health care facilit*" OR "aged care facilit*" OR "institut* care" OR "institut* care setting*" OR "institut* care facilit*")
S4	noft(experience* OR encounter* OR perspect* OR percept* OR opinion* OR attitud* OR view* OR viewpoint* OR standpoint* OR feel* OR belief* OR thought* OR support* OR need* OR requir* OR demand* OR expect* OR wish*)
S5	1 AND 2 AND 3 AND 4
S6	ab(((semi-structured or semistructured or unstructured or informal or in-depth or indepth or "face to face" or face-to-face or structured or guide) and (interview* or discussion* or questionnaire*)) or "focus group*" or observ* or fieldwork or "field work" or "key informant") OR ti(((semi-structured or semistructured or unstructured or informal or in-depth or indepth or "face to face" or face-to-face or structured or guide) and (interview* or discussion* or questionnaire*)) or "focus group*" or observ* or fieldwork or "field work" or "key informant")
S7	ab("qualitative research" or "qualitative study" or qualitative or narrat* or ethnograph*

	or phenomeno* or IPA or "interpretative phenomenological analysis" or "grounded theory" or hermeneutic* or "mixed method*" or mixed-method* or "multi method*" or multi-method*) OR ti("qualitative research" or "qualitative study" or qualitative or narrat* or ethnograph* or phenomeno* or IPA or "interpretative phenomenological analysis" or "grounded theory" or hermeneutic* or "mixed method*" or mixed-method* or "multi method*" or multi-method*)
S8	6 OR 7
S9	5 AND 8 AND la.exact(“English”)

Table8. Scoups

#	Searches
1	TITLE-ABS-KEY (dement* OR alzheimer* OR "alzheimer disease" OR ad OR "vascular dementia" OR "lewy body disease" OR "frontotemporal dementia" OR "frontotemporal lobar degeneration" OR "multi infarct dementia" OR "multi-infarct dementia" OR "mixed dementia")
2	TITLE-ABS-KEY ("nursing home personnel*" OR "nursing home employee*" OR "nursing home staff*" OR "nursing home care staff*" OR "nursing home assistant*" OR "nursing home caregiv* staff*" OR "care home staff*" OR caregiv* OR carer* OR "care giv*" OR "care staff*" OR careworker* OR "care worker*" OR nurs* OR "registered nurse*" OR rn OR "licen?ed practical nurse*" OR lpn OR "nurs* staff*" OR "nurs* aid*" OR "care aid*" OR "nurs* assistant*" OR "care attendant*" OR "nurs* auxilliar*" OR "support worker*" OR "healthcare worker*" OR "healthcare assistant*" OR "healthcare staff*" OR "health personnel*" OR "health care personnel*" OR "health care assistant*" OR "healthcare professional*" OR staff* OR "support staff*" OR worker* OR employee* OR practitioner* OR personnel* OR manager* OR leader*)
3	TITLE-ABS-KEY ("nursing home*" OR "nursing facilit*" OR "skilled nursing facilit*" OR "resident* facilit*" OR "resident* home*" OR "care home*" OR "long term care" OR "long-term care" OR "long term care setting" OR "long term care institution*" OR "long term care facilit*" OR "long-term care setting*" OR "long-term care institution*" OR "long-term facilit*" OR "resident* care institution*" OR "care facilit*" OR "health care facilit*" OR "aged care facilit*" OR "institut* care" OR "institut* care setting*" OR "institut* care facilit*")
4	TITLE-ABS-KEY (experience* OR encounter* OR perspect* OR percept* OR opinion* OR attitud* OR view* OR viewpoint* OR standpoint* OR feel* OR belief* OR thought* OR support* OR need* OR requir* OR demand* OR expect* OR wish*)
5	#1 AND #2 AND #3 AND #4
6	(ABS (((semi-structured OR semistructured OR unstructured OR informal OR in-depth OR indepth OR "face to face" OR face-to-face OR structured OR guide) AND (interview* OR discussion* OR questionnaire*)) OR "focus group*" OR observ* OR

	fieldwork OR "field work" OR "key informant") OR TITLE (((semi-structured OR semistructured OR unstructured OR informal OR in-depth OR indepth OR "face to face" OR face-to-face OR structured OR guide) AND (interview* OR discussion* OR questionnaire*)) OR "focus group*" OR observ* OR fieldwork OR "field work" OR "key informant"))
7	(ABS ("qualitative research" OR "qualitative study" OR qualitative OR narrat* OR ethnograph* OR phenomeno* OR ipa OR "interpretative phenomenological analysis" OR "grounded theory" OR hermeneutic* OR "mixed method*" OR mixed-method* OR "multi method*" OR multi-method*) OR TITLE ("qualitative research" OR "qualitative study" OR qualitative OR narrat* OR ethnograph* OR phenomeno* OR ipa OR "interpretative phenomenological analysis" OR "grounded theory" OR hermeneutic* OR "mixed method*" OR mixed-method* OR "multi method*" OR multi-method*))
8	#6 OR #7
9	#5 AND #8
10	#9 AND (LIMIT-TO (LANGUAGE , "english"))

Table9. Web of Science core collection

#	Searches
1	dement* OR alzheimer* OR "alzheimer disease" OR AD OR "vascular dementia" OR "lewy body disease" OR "frontotemporal dementia" OR "frontotemporal lobar degeneration" OR "multi infarct dementia" OR "multi-infarct dementia" OR "mixed dementia" (Topic)
2	"nursing home personnel*" OR "nursing home employee*" OR "nursing home staff*" OR "nursing home care staff*" OR "nursing home assistant*" OR "nursing home caregiv* staff*" OR "care home staff*" OR caregiv* OR carer* OR "care giv*" OR "care staff*" OR careworker* OR "care worker*" OR nurs* OR "registered nurse*" OR RN OR "licen?ed practical nurse*" OR LPN OR "nurs* staff*" OR "nurs* aid*" OR "care aid*" OR "nurs* assistant*" OR "care attendant*" OR "nurs* auxilliar*" OR "support worker*" OR "healthcare worker*" OR "healthcare assistant*" OR "healthcare staff*" OR "health personnel*" OR "health care personnel*" OR "health care assistant*" OR "healthcare professional*" OR staff* OR "support staff*" OR worker* OR employee* OR practitioner* OR personnel* OR manager* OR leader* (Topic)
3	"nursing home*" OR "nursing facilit*" OR "skilled nursing facilit*" OR "resident* facilit*" OR "resident* home*" OR "care home*" OR "long term care" OR "long-term care" OR "long term care setting" OR "long term care institution*" OR "long term care facilit*" OR "long-term care setting*" OR "long-term care institution*" OR "long-term facilit*" OR "resident* care institution*" OR "care facilit*" OR "health care facilit*" OR "aged care facilit*" OR "institut* care" OR "institut* care setting*" OR "institut* care facilit*" (Topic)
4	experience* OR encounter* OR perspect* OR percept* OR opinion* OR attitud* OR

	view* OR viewpoint* OR standpoint* OR feel* OR belief* OR thought* OR support* OR need* OR requir* OR demand* OR expect* OR wish* (Topic)
5	#1 AND #2 AND #3 AND #4
6	((semi-structured or semistructured or unstructured or informal or in-depth or indepth or "face to face" or face-to-face or structured or guide) and (interview* or discussion* or questionnaire*)) or "focus group*" or observ* or fieldwork or "field work" or "key informant" (Title) or ((semi-structured or semistructured or unstructured or informal or in-depth or indepth or "face to face" or face-to-face or structured or guide) and (interview* or discussion* or questionnaire*)) or "focus group*" or observ* or fieldwork or "field work" or "key informant" (Abstract)
7	"qualitative research" or "qualitative study" or qualitative or narrat* or ethnograph* or phenomeno* or IPA or "interpretative phenomenological analysis" or "grounded theory" or hermeneutic* or "mixed method*" or mixed-method* or "multi method*" or multi-method* (Title) or "qualitative research" or "qualitative study" or qualitative or narrat* or ethnograph* or phenomeno* or IPA or "interpretative phenomenological analysis" or "grounded theory" or hermeneutic* or "mixed method*" or mixed-method* or "multi method*" or multi-method* (Abstract)
8	#6 OR #7
9	#5 AND #8
10	#9 AND English (Languages)
