# Supplementary material for: The experience of long-term care staff caring for people with dementia in low- and middle-income Countries (LMICs): A qualitative evidence synthesis
Source: Dementia (London). 2025 May 28;25(1):173–91. doi: 10.1177/14713012251346597 (PMC12701081; doi:10.1177/14713012251346597)
Supplement: Supplemental Material - The experience of long-term care staff caring for people with dementia in low- and middle-income Countries (LMICs): A qualitative evidence synthesis [file sj-pdf-2-dem-10.1177_14713012251346597.pdf]

Appendix 2 Characteristics of the included studies

	Author/ year/ country	Study Aim/Objectives	Methodology or study design/ data collection/ data analysis	Contributing participants (gender/age/education years/ work experience)	Care setting	Key Findings/themes
1.	de Melo, R. C. 2023 Brazil	To explore long-term care facility (LTCF) staff's perspectives on the care of persons living with dementia (PLWD) during the COVID-19 pandemic in São Paulo State, Brazil.	An exploratory qualitative study/ online survey with close- and open-ended questions; open-ended questions were asked in each section to simulate an interview- guided experience/ thematic analysis	Twenty-four participants from seven LTCFs in São Paulo State, Southeast region, were invited to participate. LTCF workers whose jobs were directly related to the care of residents to participate in the current study.<br><br>The majority were paid caregivers (50%), followed by licensed practical nurses (LPNs) (16.7%). In addition, registered nurses (RNs), social workers, and managers comprised 8.3% of respondents. Physical therapists and physicians comprised only 4.2% of participants. Approximately 58% of respondents had been working in LTCF for >3 years, whereas 21% had worked in this sector for <1 year or between 1 and 3 years.	Seven LTCFs located in the municipalities of São Paulo, Botucatu, and Ourinhos agreed to participate in the study.<br><br>The majority (57.1%) of LTCFs were for-profit and the number of residents ranged between seven and 77, with an average of 28.6 (*SD* = 22.5) residents per institution. In regard to staff composition, all (100%) LTCFs had paid caregivers, followed by RNs (85.7%), physical therapists (85.7%), licensed practical nurses (LPNs; 71.4%), physicians (71.4%), social workers (57.1%), and dieticians (24.6%).	Results highlight concerns about challenges related to following precautionary measures and the negative effects of social distancing on PLWD. Aspects related to workforce and staffing and person-centered care approaches were recognized by staff as important to provide good care for PLWD. Four themes identified: theme 1: COVID-19 precautionary measures; theme 2: COVID-19 impacts on residents; theme 3: COVID-19 impacts on care and theme 4: WE-THRIVE core Domains.
2.	Jiang, J. 2022	To address the need	Qualitative	20 facility directors	Faith-based nursing homes	Four primary challenges were

	Author/ year/ country	Study Aim/Objectives	Methodology or study design/ data collection/ data analysis	Contributing participants (gender/age/education years/ work experience)	Care setting	Key Findings/themes
	China	and fill a knowledge gap in understanding challenges and coping strategies from the perspectives of nursing home leadership.	research/ semi structured interview via phone or in-person/ thematic analysis	employed by faith-based nursing homes across 12 provinces in China Among 20 participants, there were 8 males and 12 females. The majority were between ages 41 and 60. Mean ages of participants is 49.11 (+-10.3). About 55.0% of the participants had been nursing home directors for more than four years. The average year of work as a director is 4.95 years (+-3.69). Half of them participated in religious activities every day; 90.0% of the participants were Protestant, 5.0% were Catholic, and 5.0% were non-religious.	across 12 provinces in Northern, Southern, middle, and Western China, including rural and urban areas. The faith-based nursing homes included in this study were all administered by the Amity Foundation, a Chinese non-governmental organization established in 1985 by Chinese Protestant leaders. Approximately 35% had less than ten full-time nursing staff, and 65.0% had caregiver-to-resident ratios of 1 to 5 or better.	identified, including recruiting and retaining nursing staff, funding, lacking governmental support and discord with family members. The coping strategies included using external resources, incorporating religious beliefs, teamwork, rewarding performance and improving staff skills and empathy.
3.	Shrestha, S. 2022 Nepal	To explore and describe critical challenges in current dementia care practice as perceived by healthcare professionals (HCPs) in old age homes (OAHs) in	An exploratory hermeneutic design/ employing qualitative interviews with HCPs/ In-depth interviews were chosen as data collection tools/	Work experience: NA Convenience sampling was chosen. With the assistance of the OAH managers, eleven HCPs, that is two nurses from government OAH, one nurse from semi-private OAH, and six nurses and	Residing in an OAH often is the only option for older people Government OAHs are funded by the government and by donations from various voluntary organisations and individuals in Nepal and abroad. Basically,	The analysis showed that HCPs found limited educational training in dementia-specific care to be a critical challenge leading to reduced quality in caregiving practice. Insufficient HCP competence in dementia-specific care undermined adequate coping with residents' cognitive

	Author/ year/ country	Study Aim/Objectives	Methodology or study design/ data collection/ data analysis	Contributing participants (gender/age/education years/ work experience)	Care setting	Key Findings/themes
		Kathmandu, Nepal.	analysing qualitative data under hermeneutical circular process	two physio- therapists from private OAHs.	government OAHs provide housing, sanitation, clothing, healthcare, pilgrimage, religious and entertainment programmes and organising funerals and traditional death anniversary celebrations (Shrestha, 2006). Private OAH admission is usually paid by residents' families, and these OAHs have better healthcare services, dwelling units and other facilities (Mishra & Chalise, 2018).	disturbances and the behavioural and psychological symptoms in dementia (BPSD). Poor HCP/medical doctor (MD)-to-patient ratio was perceived as a critical challenge preventing proper diagnostic examination, treatment and dementia-specific care practice.
4.	Md Hussin, N. S. 2023 Malaysia	To explore the current practice of managing BPSD and to identify the potential strategies and challenges in executing non-pharmacological interventions in secondary care facilities in Malaysia.	A qualitative study/ in-depth face-to-face interview/ thematic analysis	12 caregivers and 11 people with dementia (PWD) were included.	A geriatrician (Tan KM) provided suggestions for eligible care centres according to the criteria. Seven secondary care facilities for PWD — comprising five nursing homes and two day care centres in the urban area of Klang.	The non-pharmacological interventions being practised in secondary care facilities included music therapy, exercise and motor rehabilitation, and cognitive games and activities. Other non-pharmacological interventions were reminiscence therapy, massage and touch therapy, arts and craft, acupuncture, pet therapy and coconut oil consumption. The ten themes that emerged comprised strategies and barriers related to three main categories:

	Author/ year/ country	Study Aim/Objectives	Methodology or study design/ data collection/ data analysis	Contributing participants (gender/age/education years/ work experience)	Care setting	Key Findings/themes
						person-centred care, social interaction and external roles. Collaborative care from the care providers and family members was found to be an important facilitating factor. The lack of family support led to care providers carrying additional workload beyond their job scope. Other barriers to non-pharmacological interventions were cultural and language differences between the care providers and PWD, inadequate staff numbers and training, and time constraints.
5.	Siewert, J.S. 2021 Brazil	To understand nursing workers' experiences with the nursing care provided to elderly individuals with dementia living in a long-term care facility.	A comprehensive qualitative study / Data was collected using questionnaires, field diaries, individual interviews, and participatory observation / Thematic content analysis was used to interpret content.	Thirteen nursing technicians and one nurse participated, totalling 14 professionals working in the LTCF. Among the participants who met the inclusion and exclusion criteria, six were women, and eight were men. Two were aged between 25 and 29; six were aged from 30 to 39 years old; four were aged from 40 to 49 years old, and two between 50 and 59 years old. Regarding time since	This comprehensive qualitative study was conducted in a long-term care facility located in Joinville, Santa Catarina, Brazil. At the time of data collection, this LTCF had one nurse and 14 nursing technicians. This LTCF was chosen due to the proximity between the researcher and the facilty's team and the large nursing staff. Its 48 beds were occupied at the time of data collection. Of these,	Three themes emerged from data analysis: "Knowledge acquired with practice and gap existing in the care provided to institutionalized elderly individuals with dementia"; "Individualized care provided to elderly individuals and bonding"; and "Conflicts the nursing staff faces when reconciling care needs, the time available, and the facility's routines". These themes originated the central theme: Meanings assigned to the nursing care provided to institutionalized elderly individuals with dementia. By sharing information regarding care

	Author/ year/ country	Study Aim/Objectives	Methodology or study design/ data collection/ data analysis	Contributing participants (gender/age/education years/ work experience)	Care setting	Key Findings/themes
				graduation, six had graduated up to four years ago, five had graduated up to nine years ago, and three workers had graduated up to 14 years ago. Regarding their experience in that specific LTCF and in this type of care, most (n=9) worked in the facility for four years, and most (n=9) had up to four years of experience providing care to elderly individuals.	15 elderly individuals had a medical diagnosis of dementia. The facility has one nursing ward, where five older women in advanced stages of dementia and entirely dependent for daily living activities were allocated.	actions that obtained positive results, the staff perceived the importance of an individualized approach and of establishing bonds as a therapeutic process. Gaps were identified in the professionals' knowledge regarding how to provide hygiene care when individuals become aggressive, agitated, or resist care.
6.	Strom, B.S. 2021 India	To explore the knowledge about and attitude towards dementia among nursing staff working in residential care facilities for older people in India.	An explorative and descriptive qualitative design was used/ Two semi- structured focus group interviews were conducted/ Qualitative content analysis was used.	Twelve participants, i.e., 8 nurses and 4 care assistants, all female, with an average age of 45.5 years (range 22–82 years), were recruited to take part in the focus group interviews.<br>The participants' experience working with older people ranged from 5 months to 20 years, with an average of 4.5 years.	The participants represented 6 residential care facilities for older people in different parts of India run by Catholic religious orders.	The participants highlighted the following 3 dimensions in relation to their knowledge of and attitudes toward dementia in residential care facilities in India: (1) people with dementia – a walking mystery; (2) we need to go along with them, but it is challenging; and (3) if we know, we can care for them in a better way.
7.	van Wyk, A. 2017 South Africa	Explore the possible unmet needs of staff	Qualitative interpretative	Their mean age was 48 years (range 32–66) and	They were interviewed across the four care homes	Findings reflected the literature with regard to examples of

	Author/ year/ country	Study Aim/Objectives	Methodology or study design/ data collection/ data analysis	Contributing participants (gender/age/education years/ work experience)	Care setting	Key Findings/themes
		working with residents with dementia in long-term care facilities (care homes) and what they might regard as effective support for themselves; Explore which behaviours of care home residents with dementia are regarded by care home staff as challenging to deal with and how they manage such behaviour; Hear from staff how they learn to deal with distressing or challenging behaviour.	studies / semi-structured interviews/ thematic analysis	participants had an average of 15 years' care home work experience. No male members of care staff were employed by any of the care homes. Participants were either English or Afrikaans speaking and interviews were conducted in the participant's language of choice. Participants were all day staff who did not rotate with night duty staff.	(referred to as A, B, C or D). The care homes participating in this study were from one province which is not necessarily representative of all South Africa's care homes.	behavioural symptoms of people with dementia that staff find challenging to manage. Overall, the majority of staff reported holding positive feelings about working with people with dementia. All preferred interpersonal approaches to manage distressed behaviour above medication although a small minority noted the use of medication in some cases. Dementia training was considered by most participants as an unmet need.
8.	Wang, J. 2022 China	to understand person-centered care (PCC) from the perspectives of care aides, and to identify implications to facilitate and sustain their efforts in	A descriptive qualitative methodology grounded in a naturalist philosophy because our goal was to be "data-near" /	40 care aides from 5 nursing homes in China/ Characteristics & Participants: Age, mean (SD) 51(5.6), female (%) 90, educational Level (%) : College and higher High school 25, Middle	More than 80% of long-term care (LTC) facilities are non-profits and their service charges are comparable. All the non-profit facilities are eligible for government subsidies (such as rental fee	We found that the seemingly beneficial mental models used by care aides in their work can hinder them from playing a more adaptive role in tailoring their care to the needs of older residents. Infantilizing older residents with dementia and labeling them using

	Author/ year/ country	Study Aim/Objectives	Methodology or study design/ data collection/ data analysis	Contributing participants (gender/age/education years/ work experience)	Care setting	Key Findings/themes
		providing quality care for older residents with dementia. Therefore, we conducted a qualitative study to understand the relevance of person-centered dementia care in nursing homes from the perspectives of nursing care aides in a low-resource context.	interviewed 40 care aides from 5 nursing homes in China. We investigated the information on the aides' behaviors, interactions, and communication at the point of care within the organizational context as a supplement to the interview data regarding their perceptions of PCC / applied conventional content analysis to examine the interview data and field notes.	school 55, Elementary school 20 Household registry (Hukou): Urban 25, Rural 75<br>Months working in the facility (SD) 31(4.5)<br>Living arrangement: Outside of nursing home 55, Live in the nursing home 45.	reductions) because their pricing is monitored by the Shanghai Municipal Price Bureau and they are recognized as non-profit organizations.5We selected five non-profit LTC facilities with dementia care units in Shanghai through our connections and research collaborations with professional committees, oversight agencies, and LTC facilities by taking into account characteristics such as location, size, ownership, charges, and the availability of on-site health care clinics. These LTC facilities have established close ties with hospitals that provide regular health care services to older residents. All care aides included in this study were from the five non-profit facilities.	mother wit can prevent meaningful, equal, and person-centered conversations between both parties. Care aides do not have regular formal interactions and sensemaking with nurses and other professionals in nursing homes. Increasing interactions and communication between care aides and health care professionals in nursing homes can lead to insight for changing the approach to in-service training to achieve better acceptance by care aides.
9.	Yang, Q. 2024 China	to elucidate the methodologies employed by nursing	a phenomenological approach / Data was accrued	The study encompassed interviews with 17 nursing assistants, comprised of 2	A prominent public non-profit nursing facility in Guangzhou, China. The	Data analysis yielded four emergent themes: 1) Perception of pain; 2) Strategies for coping with

	Author/ year/ country	Study Aim/Objectives	Methodology or study design/ data collection/ data analysis	Contributing participants (gender/age/education years/ work experience)	Care setting	Key Findings/themes
		assistants in the identification and management of pain among dementia patients. This will include an exploration into their subjective experiences and the obstacles they encounter in executing this critical facet of care.	through individual, face-to-face semi-structured interviews / thematic analysis	males and 15 females. Participants' ages varied between 22 and 49 years, with a mean age of 40.3 years. Duration of experience in dementia care spanned from 1 to 28 years, averaging at 9.8 years.	institution comprises 3,800 beds dispersed over three distinct campuses, including two specialized dementia care centers housing over 400 residents.	pain; 3) Emotional and psychological responses to pain-related caregiving; and 4) Challenges and needs in pain-related caregiving. Nursing assistants play a unique and crucial role in the identification of pain, reporting and providing feedback to healthcare professionals, and implementing non-pharmacological interventions.
10.	Yektatalab, S. 2012 Iran	this study was designed to explore the perceptions of Iranian caregivers about caring Alzheimer patients in the elderly care homes.	This qualitative study used the principles of inductive content analysis / Two focus groups and 4 qualitative interviews were conducted. The participants of the focus groups were formal caregivers who had not been educated on nursing or gerontology principles. To complete the data, four other	14 key informants (10 women and 4 men, between 25-35 years of age) who had been working in elderly care homes, caring for the elderly with Alzheimer disease for about 1-11 years (Mean=30 months), were selected by purposive sampling method. The caring experience and ability of transferring their experience to others were the main criteria for selection of the participants. To complete	There were five elderly care homes in Shiraz, southwestern Iran. Two elderly care homes which admitted Alzheimer disease patients were purposefully selected.	Nearly, 800 initial codes were extracted and categorized into 3 groups of "multidimensional care", "going along with the patients" and "need to be professional" and 12 subcategories. Although several aspects of care were mentioned by the participants but the main aspect was physical care. Infantilizing the patients was the main feature of care and caring personality was an important characteristic of caregivers.

	Author/ year/ country	Study Aim/Objectives	Methodology or study design/ data collection/ data analysis	Contributing participants (gender/age/education years/ work experience)	Care setting	Key Findings/themes
			interviews were performed with head nurses and supervisors.	the data, four other interviews were performed with head nurses and supervisors. Two of them were the holders of bachelor in nursing and two in psychology.		
11.	Zhao, Y. 2021 China	This study is part of a larger study which aims to develop a culturally ap- propriate dementia care education programme for care home staff in China grounded on service gaps identified in the qualitative findings of this study.	An exploratory qualitative study, including participant observations, review of residents' care records and in-depth interviews with care home staff, was conducted / Data including care records, field notes and interview transcripts were analysed using qualitative content analysis described by Graneheim and Lundman (2004).	Amongst the 15 participants, four were nurse managers, four were nurses and seven were care assistants. Only one of them was male. Their mean age was 41.5 years (*SD* = 11.9). All nurses and nurse managers completed college education in nursing, except one nurse manager who had a bacca- laureate education. The education level of care assistants was primary or junior high school. Their mean working experience related to dementia care was 3.1 years (*SD* = 2.0), ranging from 1 to 9 years. The inclusion criteria were	The study was conducted at four care homes with different levels of scale and resources at different locations in Nanjing, China. Around one-thirds residents had a diagnosis of dementia, ranging from early to late-stage, in each participating care home.	Four categories about dementia care practices in care homes were identified: (a) care environment (hospital-like layout, inappropriate lighting, environmental noise, inappropriate use of colour and unclear signage), (b) care culture (being medical-oriented, overlooking individual uniqueness and privacy), (c) attitudes towards dementia (treating as children, being authoritative, adopting punitive approaches, trying to respect the residents and having a positive learning attitude) and (d) dementia care competence (questing for specific training and resources, questing for culturally specific practices and strengthening communication with family).

	Author/ year/ country	Study Aim/Objectives	Methodology or study design/ data collection/ data analysis	Contributing participants (gender/age/education years/ work experience)	Care setting	Key Findings/themes
				having more than 1-year working experience in dementia care and willing to give written informed consent for participating in the study. Staff members who were not involved in direct care of residents with dementia were excluded.		

List of abbreviation

BPSD, Behavioural and psychological symptoms in dementia; HCPs, Healthcare professionals; LPNs, licensed practical nurses; LTC, long-term care; LTCF, long-term care facility; MD, Medical doctor; NA, Not available; OAHs, Old age homes; HCPs, Healthcare professionals; PCC, Person-centered care; PWD, people with dementia; PLWD, persons living with dementia; RNs, registered nurses; SD, standard deviation.

Reference

Graneheim, U. H., & Lundman, B. (2004, Feb). Qualitative content analysis in nursing research: concepts, procedures and measures to achieve trustworthiness. *Nurse Educ Today, 24*(2), 105-112. https://doi.org/10.1016/j.nedt.2003.10.001

Mishra, S., & Chalise, H. (2018). Health status of elderly living in government and private old age home in Nepal. Asian Journal of Biological Sciences, 11(4), 173-178.

Shrestha, U. L. (2006). Social Welfare Centre Elderly's Home, Pashupatinath. http://globalag.igc.org/elderrights/world/2007/elderlyshome.htm
