# Supplementary material for: The experience of long-term care staff caring for people with dementia in low- and middle-income Countries (LMICs): A qualitative evidence synthesis
Source: Dementia (London). 2025 May 28;25(1):173–91. doi: 10.1177/14713012251346597 (PMC12701081; doi:10.1177/14713012251346597)
Supplement: Supplemental Material - The experience of long-term care staff caring for people with dementia in low- and middle-income Countries (LMICs): A qualitative evidence synthesis [file sj-pdf-3-dem-10.1177_14713012251346597.pdf]

Appendix 3. CASP Quality Appraisal Scoring Tables for This Review

Quality questions: Were the following areas adequately addressed or appropriately applied?
0= not or inadequately addressed/applied; 1= adequately addressed/applied; 2= well addressed/applied
Overall Score: 0-7= low quality; 8-14= moderate quality; 15-20= high quality.

Table 1. CASP Quality Appraisal Scoring Tables for Included Studies

	Author/ year	Q1.	Q2.	Q3.	Q4.	Q5.	Q6.	Q7.	Q8.	Q9.	Q10.	Score (n=20)
1.	de Melo, R. C. 2023	2	1	2	2	2	0	2	2	2	2	17
2.	Jiang, J. 2022	2	2	1	1	2	0	2	2	2	2	16
3.	Md Hussin, N. S. 2023	2	2	2	2	2	0	2	2	2	2	18
4.	Shrestha, S. 2022	2	2	2	2	1	2	2	2	2	2	19
5.	Siewert, J.S. 2021	2	2	2	1	2	1	2	2	2	2	18
6.	Strom, B.S. 2021	2	2	2	1	2	0	2	2	2	2	17
7.	van Wyk, A. 2017	2	2	2	1	2	0	2	2	1	2	16
8.	Wang, J. 2022	1	2	2	2	2	0	2	2	2	2	17
9.	Yang, Q. 2024	2	2	2	2	2	1	2	2	2	2	19
10.	Yektatalab, S. 2012	2	2	2	2	2	0	1	2	2	2	17
11.	Zhao, Y. 2021	2	2	2	2	2	2	2	2	2	2	20

Q1. Was there a clear statement for the aims of the research?
Q2. Is a qualitative methodology appropriate?
Q3. Was the research design appropriate to address the aims of the research?
Q4. Was the recruitment strategy appropriate to the aims of the research?
Q5. Was the data collected in a way that addressed the research issue?
Q6. Has the relationship between the researcher and participants been adequately considered?
Q7. Have ethical issues been taken into consideration?
Q8. Was the data analysis sufficiently rigorous?
Q9. Is there a clear statement of findings?
Q10. How valuable is the research?
